# An Elegant Sticking Plaster

**Published:** 1844-01

**Authors:** 


*An Elegant Sticking Plaster.*—Black silk is strained and brushed over ten or twelve times with the following preparation:—Dissolve ℥ss of Benzoin in f℥vi of rectified spirit; in a separate vessel dissolve ℥j. of isinglass in water; strain each solution, mix them, and let the mixture rest, so that the grosser parts may subside; when the clear liquor is cold it will form a jelly, which must be warmed before it is applied to the silk. When the plaster is quite dry, in order to prevent its cracking, it is finished off with a solution of terebinth. chia. ℥iv. in tinct. benzoes f℥vj.—*Medical Times*, April 15, 1843.

